# The Resistive Index Is a Marker of Renal Function, Pathology, Prognosis, and Responsiveness to Steroid Therapy in Chronic Kidney Disease Patients

**DOI:** 10.1155/2012/139565

**Published:** 2012-12-16

**Authors:** Kikuno Hanamura, Akihiro Tojo, Satoshi Kinugasa, Kensuke Asaba, Toshiro Fujita

**Affiliations:** Division of Nephrology and Endocrinology, University of Tokyo, 7-3-1 Hongo, Bunkyo-ku, Tokyo 113-8655, Japan

## Abstract

To evaluate the significance of the renal resistive index (RI) as a noninvasive marker of renal histological damage and a prognostic indicator, we examined RI by Doppler ultrasonography in 202 chronic kidney disease (CKD) patients who underwent renal biopsy. RI increased as the CKD stage progressed and correlated with age, systolic blood pressure, estimated glomerular filtration rate (eGFR), and renal histological changes, including glomerulosclerosis, arteriolosclerosis, and tubulointerstitial damage. Prognostic evaluation with a median follow-up period of 38.5 months revealed that patients with RI ≥ 0.7 (high RI group, *n* = 39) had significantly poorer renal survival than those with RI < 0.65 (normal RI group, *n* = 120) and 0.65 ≤ RI < 0.7 (high-normal RI group, *n* = 43). The patients in the high-normal RI group showed good response to steroids. However, in the high RI group, steroid therapy did not significantly improve renal survival. Of the clinical indices studied, RI ≥ 0.7, hypertension, proteinuria, and low eGFR at diagnosis were independent risk factors for worsening renal dysfunction. In conclusion, RI in CKD patients was considered as a marker of renal function, histological damage, and renal prognosis, and a possible determinant of indication for steroids.

## 1. Introduction

Chronic kidney disease (CKD) is a known risk factor for end-stage renal disease (ESRD) and cardiovascular diseases. Early detection and making adequate therapeutic decisions are essential for the medical care of CKD. Doppler ultrasonography is a noninvasive method widely used in clinical practice for CKD patients. It can detect not only renal macroabnormalities but also changes in the renal vasculature and blood flow. The resistive index (RI) is commonly used as an index of intrarenal arterial resistance. RI increases in various kidney diseases [1–9], and previous studies have shown the associations of RI with renal function and patient prognosis [4, 10–18]. In a clinical setting, we sometimes experience patients with increased RI, indicating a poor response to steroid therapy and progression to ESRD. However, to our knowledge, whether increased RI affects responsiveness to steroids remains unknown. Moreover, the effects of moderately elevated RI within normal limits on renal prognosis have not yet been clarified.

 The relationship between renal histological changes and RI has been investigated previously. Glomerulosclerosis (GS) [19], tubulointerstitial (TI) damage [10, 19, 20], and vascular lesions [10, 14, 19] have been reported to correlate with an increase in RI. However, the results were not always consistent [15], and because past studies regarding renal histology have often investigated small populations, they have not studied patients from all stages of CKD, and the associations between RI and renal histological changes regarding their locus and severity have not been sufficiently elucidated.

 This study aimed to evaluate the significance of RI as a non-invasive marker of renal histological damage, and also to study the effect of increased RI on renal prognosis and responsiveness to steroid therapy in order to determine whether it is useful in making therapeutic decisions in the clinical care of CKD patients.

## 2. Patients and Methods

### 2.1. Patients and Clinical Evaluation

A total of 202 consecutive Japanese CKD patients diagnosed by renal biopsy from December 2001 to March 2010 at our department were examined. The CKD diagnostic criteria were based on the guidelines proposed by the Kidney Disease Outcomes Quality Initiative (K/DOQI) of the National Kidney Foundation (2002) [21], and the classifications were made as follows: stage 1, estimated glomerular filtration rate (eGFR) > 90; stage 2, eGFR = 60–89; stage 3, eGFR = 30–59; stage 4, eGFR = 15–29; and stage 5, eGFR < 15 or dialysis. eGFR was calculated using the revised equation for Japanese patients, that is, eGFR (mL/min/1.73 m^2^) = 194 × [serum creatinine (Cr) (mg/dL)]^−1.094^  × (age)^−0.287^ (×0.739 if female) [22, 23]. For each patient, the age, sex, systolic blood pressure, urinary protein, serum creatinine level, and eGFR at renal biopsy were recorded. The study was conducted in accordance with the Declaration of Helsinki, and written informed consent was obtained from all the patients with the approval of the Research Ethics Committee of the University of Tokyo Hospital (no. 1807).

### 2.2. Ultrasonographic Measurement

Ultrasound testing was performed on the day before renal biopsy by the same operator (A.T.) for all the patients. In the maximum long-axis section images, the largest diameter and width of each kidney were measured, usually in a prone position. The renal cortex area was calculated using the following formula: Renal cortex area (cm^2^) = *πAB*/4 − *πab*/4, where *A* = renal length (cm), *B* = renal width (cm), *a* = length of the central echo complex (cm), and *b* = width of the central echo complex (cm). The SONOS5500 (Agilent Technologies, CA, USA) or NemioXG (Toshiba Medical Systems, Tochigi, Japan) ultrasound device and a 3.5-MHz probe were used to obtain images for RI measurement. In each patient, RI at the interlobular or arcuate artery near the border of the central echo complex was measured three times in the upper, middle, and lower portions of the kidney in a supine position and was averaged for each kidney. The mean RI value of both kidneys was used for analysis. The normal range of RI is 0.5–0.7 [4, 10, 15].

### 2.3. Histological Evaluation

The renal biopsy samples were evaluated for the severity of GS, arteriolosclerosis (AS), and TI damage based on five-level scoring systems [24, 25]. In brief, GS and AS scores were evaluated in periodic acid-Schiff (PAS-) stained sections, and the GS score was defined as follows: 0, normal GS; 1, matrix expansion or GS < 25%; 2, GS = 26%–50%; 3, GS = 51%–75%; and 4, GS > 75%. The AS score was defined as follows: 0, normal; 1, medial thickening; 2, segmental hyalinosis; 3, global hyalinosis; and 4, luminal occlusion with thrombus or infiltrating cells. The TI score was assessed in azan- or periodic acid-methenamine silver (PAM-) stained sections and was defined as follows: 0, normal; 1, mild fibrosis around the vasculature; 2, mild fibrosis around tubules; 3, moderate fibrosis with tubular casts or tubular damage; and 4, severe fibrosis with cell infiltration. The average score of the entire area of the biopsy sample was calculated for each histological component in each patient.

### 2.4. Prognostic Evaluation

Renal prognosis was evaluated by the combined endpoint of either doubling of the serum Cr level or ESRD necessitating regular dialysis. Followup was discontinued when regular dialysis became necessary because of a decline in renal function.

### 2.5. Statistical Analysis

All data are shown as the mean ± standard error of the mean (SEM). The box plots show the sample minimum; lower, median, and upper quartiles; and sample maximum. Correlations between variables were evaluated by Spearman's rank correlation test. Comparisons between the two groups were based on two-sided *t*-tests and the *χ* ^2^-test when appropriate. For comparisons of the variables among the different RI groups, the Kruskal-Wallis test was performed using the Steel-Dwass procedure. The impact of clinical and histological factors on ultrasonographic measurements was evaluated by stepwise multivariate regression analysis. The efficacy of the prognostic factors was examined using the Cox proportional hazard model, and the cut-off values for each variable were determined by the sensitivity and specificity obtained from a receiver operating characteristic curve. Comparison of survival from the endpoint was performed by Kaplan-Meier analysis and a log-rank test. A *P* value <0.05 was considered to be statistically significant.

## 3. Results

### 3.1. Clinical Backgrounds of the Patients

A total of 202 CKD patients were diagnosed by renal biopsy. These included 81 patients with IgA nephropathy, 26 with focal segmental glomerulosclerosis (FSGS), 24 with membranous nephropathy, 24 with minimal change diseases, 10 with diabetic nephrosclerosis, 10 with crescentic glomerulonephritis, 5 with hypertensive nephrosclerosis, 5 with lupus nephritis, 4 with interstitial nephritis, 3 with amyloidosis, 3 with hereditary nephritis, 3 with hematopoietic stem cell transplantation-related nephropathy, 2 with postinfectious acute glomerulonephritis, and 2 with membranoproliferative glomerulonephritis. There were 110 male and 92 female patients with a mean age of 48 ± 1 (range, 16–84) years and a mean systolic blood pressure of 125 ± 1 mmHg, urinary protein level of 3.8 ± 0.3 g/gCr, serum Cr level of 1.33 ± 0.09 mg/dL, eGFR of 61.8 ± 2.1 mL/min/1.73 m^2^, and RI of 0.631 ± 0.006. There were 33 patients with CKD stage 1, 75 with stage 2, 64 with stage 3, 20 with stage 4, and 10 with stage 5.

### 3.2. Ultrasonographic Measurements of Kidney Sizes

**Figure 1:**
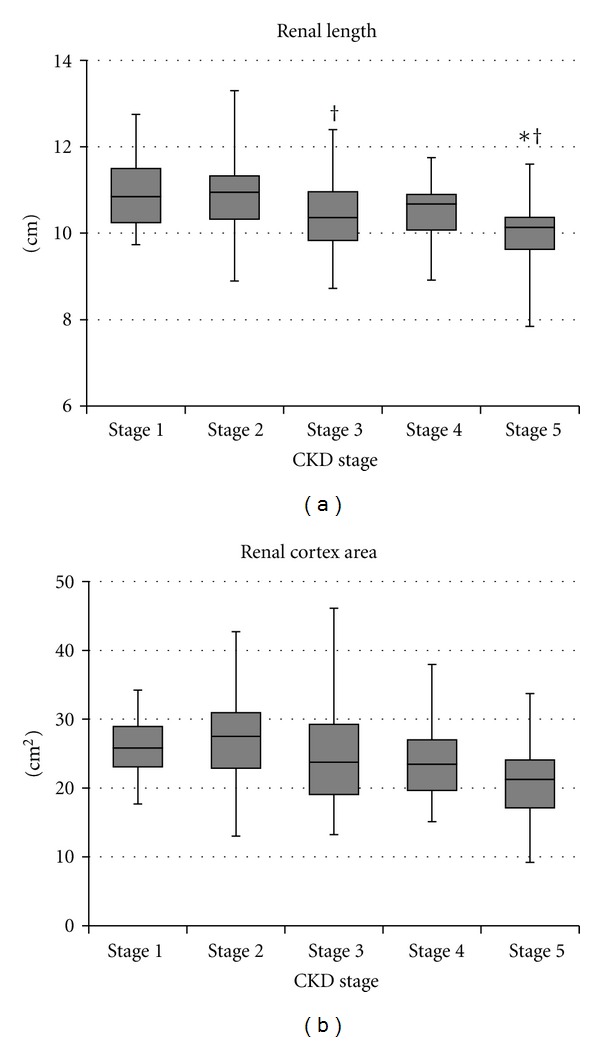
Changes in kidney sizes based on chronic kidney disease (CKD) stages. The renal length was significantly smaller in patients with CKD stage 5 than in those with CKD stage 1 or 2 (*P* < 0.01). However, in CKD stages 1 to 4, there was no clear association between the disease stage and kidney size. *P < 0.05 versus stage 1, ^†^ *P* < 0.05 versus stage 2.

The renal length was significantly smaller in patients with CKD stage 5 than in those with CKD stage 1 or 2 (Figure 1). However, in stages 1 to 4, there was no clear association between the kidney size and disease progression, and the renal length and cortex area showed a weak correlation with renal function and histological damage scores (Table 1).

### 3.3. Correlation of RI with Clinical and Histological Indices

**Figure 2:**
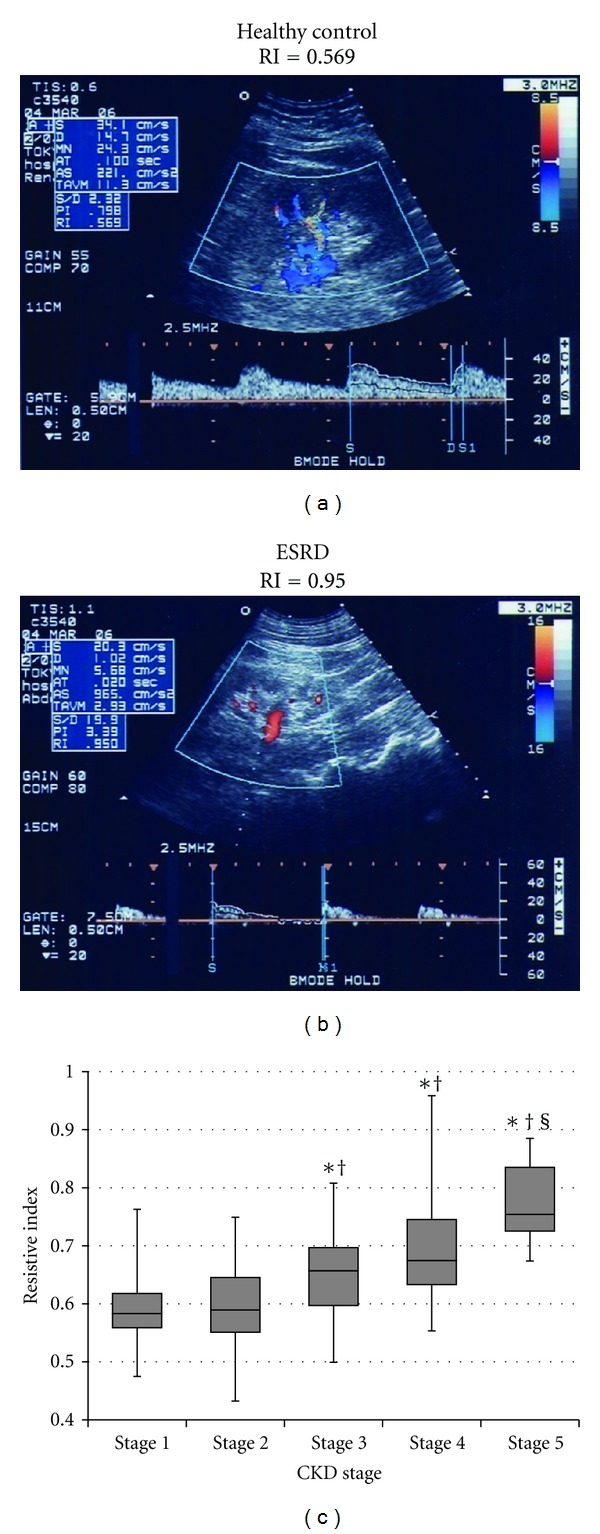
The resistive index (RI) based on CKD stages. An ultrasonographic image of a healthy control with CKD stage 1 (a) and of a patient with end-stage renal disease (ESRD) with CKD stage 5 (b). RI increased with CKD progression (*P* < 0.001) (c). *P < 0.01 versus stage 1, ^†^ *P* < 0.01 versus stage 2, ^§^ *P* < 0.01 versus stage 3.

**Figure 3:**
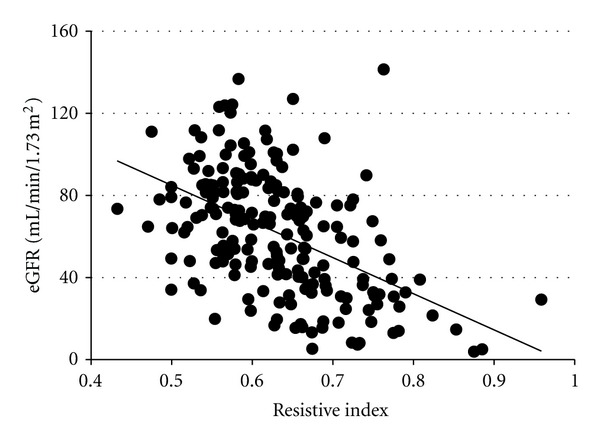
The correlation between RI and renal function. RI showed a correlation with estimated glomerular filtration rate (eGFR) (*r* = −0.52, *P* < 0.01).

**Figure 4:**
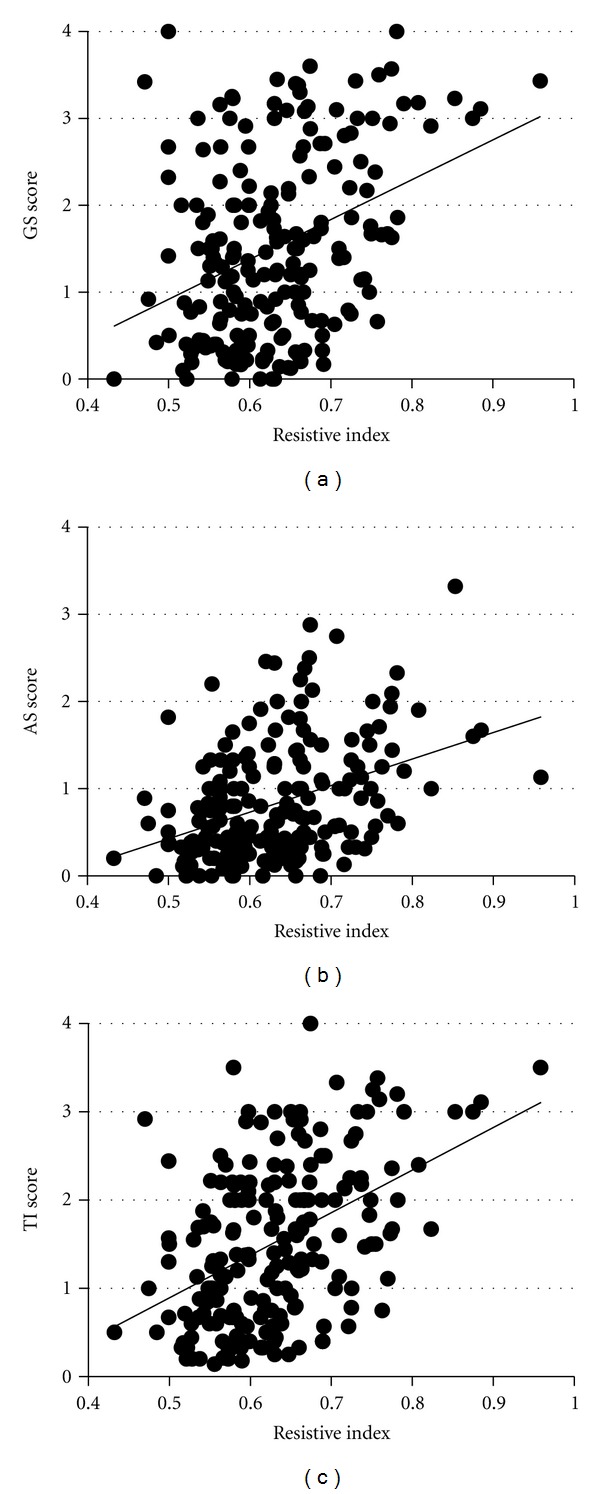
RI and histological damage scores. RI was correlated with glomerulosclerosis (GS), arteriolosclerosis (AS), and tubulointerstitial (TI) damage scores. The highest correlation was shown in TI lesions (*r* = 0.32, *P* < 0.01 for the GS score; *r* = 0.36, *P* < 0.01 for the AS score; and *r* = 0.43, *P* < 0.01 for the TI score).

In contrast, RI increased as the CKD stage progressed (Figure 2), and it was correlated with renal function (Figure 3) and histological damage scores (Figure 4), showing the best correlation with TI lesions among the three histological components (Table 1). RI was also associated with patient age and systolic blood pressure; however, it was not associated with the severity of urinary protein excretion. Stepwise multivariate regression analysis showed that patient age (*β* = 0.20, *P* < 0.01), eGFR (*β* = −0.24, *P* < 0.01), and TI score (*β* = 0.23, *P* < 0.01) were risk factors for increased RI in CKD patients (*r* ^2^ = 0.30, *P* < 0.001). RI values that best estimated CKD stages ≥4 and CKD stage 5 were 0.66 (73% sensitivity and 73% specificity) and 0.72 (80%, 88%), respectively. RI values that best estimated a GS score ≥2, an AS score ≥2, and a TI score ≥2 were 0.65 (58%, 67%), 0.66 (73%, 72%), 0.65 (63%, 72%), respectively.

### 3.4. RI and Renal Prognosis

**Figure 5:**
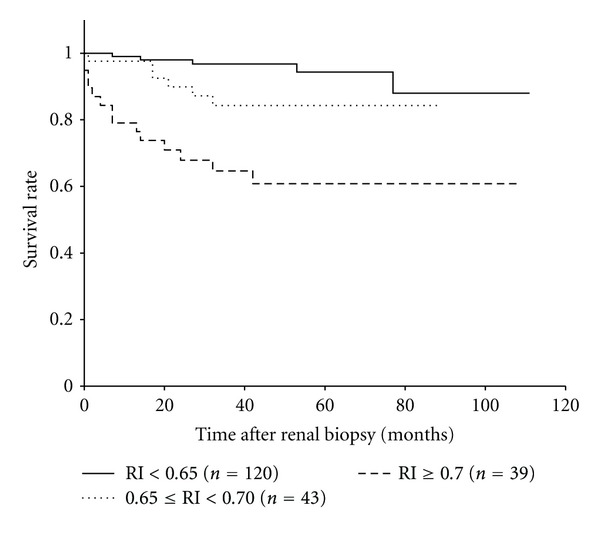
Kaplan-Meier analysis for survival from the decline in renal function by RI at renal biopsy (*n* = 202). Patients with RI ≥ 0.70 (high RI group) showed significantly poorer prognosis than those with RI < 0.65 (normal RI group) (*P* < 0.0001) or with 0.65 ≤ RI < 0.70 (high-normal RI group) (*P* = 0.02). Renal survival in the normal and high-normal RI groups did not differ significantly (*P* = 0.06). The 3-year survival rates for the normal, high-normal, and high RI groups were 0.967, 0.843, and 0.646, respectively.

All the patients were examined for renal prognosis for a median follow-up period of 38.5 (range, 1–111) months. Twenty-five patients (12.4%) showed declined renal function. Of these, 5 (2.5%) had a doubling of serum Cr levels and 20 (9.9%) progressed to ESRD. The patients were divided into three groups based on RI at renal biopsy as follows: the normal RI group (RI < 0.65, *n* = 120), high-normal RI group (0.65 ≤ RI < 0.7, *n* = 43), and high RI group (RI ≥ 0.7, *n* = 39). The patients in the normal RI group were younger and had higher eGFR and milder TI damage than those in the other two groups (Table 2). In contrast, the patients in the high RI group were more hypertensive and had more severe AS than those in the normal RI group, and they also had lower eGFR and more severe GS than the patients in the other two groups. Kaplan-Meier analysis revealed that the patients in the high RI group had significantly poorer prognosis than those in the other groups (Figure 5).

### 3.5. Risk Factors for the Worsening of Renal Function

Univariate analysis using the Cox proportional hazard model showed that age, systolic blood pressure, eGFR, urinary protein level, histological scores, and RI were effective prognostic factors for declining renal function, and the hazard ratio of the patients with RI ≥ 0.7 was 5.83 [95% confidence interval (CI), 2.65–12.85]. Multivariate analysis with stepwise selection, including all the histological parameters, revealed that patient age, urinary protein level, low eGFR, and high GS score were independent risk factors for the progression of renal dysfunction (Table 3). Evaluation of only clinical or non-invasive markers revealed that high RI at renal biopsy, high systolic blood pressure, urinary protein level, and low eGFR were independent risk factors for the progression of renal dysfunction.

### 3.6. RI and Response to Steroid Therapy

**Figure 6:**
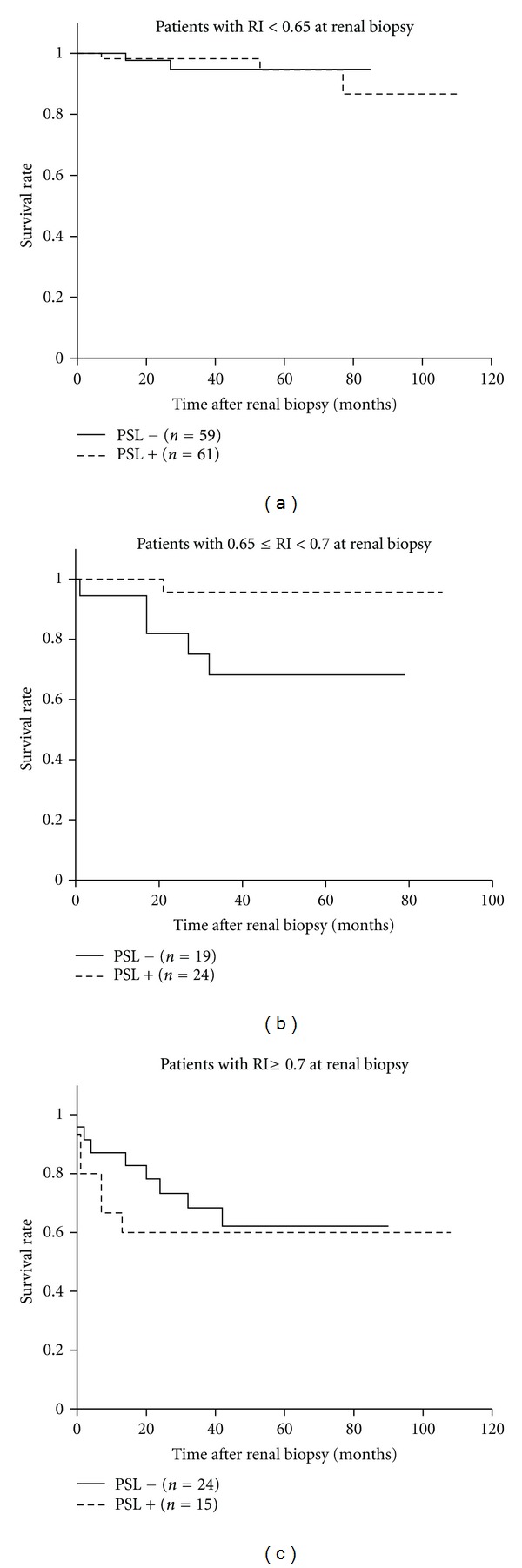
Kaplan-Meier analysis of patients with different levels of RI stratified by steroid administration. The patients in the high-normal RI group showed good response to steroids. However, in the high RI group, steroid therapy did not significantly improve renal survival. The 3-year renal survival rates with and without steroid therapy were 0.982 and 0.947, respectively, (*P* = 0.89) in the normal RI group; 0.957 and 0.682, respectively, (*P* = 0.02) in the high-normal RI group; and 0.600 and 0.684, respectively, (*P* = 0.54) in the high RI group.

Prognostic evaluation stratified by the use of steroids provided more precise information on the renal outcome in each RI group (Figure 6). In the normal RI group, despite heavy proteinuria at renal biopsy, the patients who underwent steroid therapy showed a high rate of survival similar to those who did not require steroid administration. In addition, in the high-normal RI group, the patients showed an excellent response to steroid therapy and had preferable outcomes with steroid administration. However, in the high RI group, steroid therapy did not significantly improve renal survival, suggesting poor responsiveness. Multivariate analysis with stepwise selection revealed that high RI at renal biopsy, advanced age, and glomerulosclerosis were independent risk factors for poor prognosis of the patients who underwent steroid therapy. Proportions of the patients who were administered angiotensin-converting enzyme inhibitors (ACE-I) or angiotensin II receptor blockers (ARB) were similar in the patients with and without steroid therapy (64/100 versus 68/102; *P* = 0.69).

### 3.7. Risks of High-Normal Range RI without Steroid Treatment

Renal survival of the patients in the normal and high-normal RI groups did not differ significantly in overall comparison. However, the patients in the high-normal RI group who did not undergo steroid therapy had significantly poorer prognosis than those in the normal RI group (*P* = 0.01, the log-rank test), similar to the patients in the high RI group (*P* = 0.74). For the patients in the high-normal RI group at diagnosis who did not receive steroid administration, the hazard ratio for adverse renal outcome was 6.82 (95% CI = 1.32–35.15) compared with such patients in the normal RI group.

## 4. Discussion

In the present study, we evaluated the various utilities of the resistive index, measured by Doppler ultrasonography, in CKD patients. Of the ultrasonographic indices studied, RI was the best marker of CKD stages. RI increased with the CKD stage and showed correlations with renal function and histological damage scores. In contrast, the renal length and cortex area showed only a weak association with renal function. Kidney sizes are often affected by the patients' body size [26, 27], and certain disease conditions such as diabetic nephropathy, rapidly progressive glomerulonephritis, and amyloid nephropathy may also result in kidney enlargement. Our results suggested that the indices of kidney sizes were a poor indicator of the CKD stage.

RI showed a correlation with all histological parameters, and the highest correlation was observed with TI lesions in the present study. Ikee et al. reported that AS showed the best association with elevated RI [14]. The inclusion of a younger population with relatively mild renal dysfunction in their study (mean age, 43.5 ± 16.1 years; creatinine clearance, 76.5 ± 32.1 mL/min; and RI, 0.556 ± 0.056) may have contributed to different results of their study compared with those of our study. The correlation of RI with TI damage independent of renal function was a notable finding in our study. Interstitial fibrosis with tubular atrophy and loss of capillaries are common findings in advanced kidney injuries, and TI damage is a histological parameter that best correlates with renal function [28]. Although the mechanisms by which TI damage can cause an increase in RI remain unknown, alterations in postglomerular vessels by interstitial fibrosis can cause increased resistance to renal cortical blood flow, with a subsequent reduction of glomerular perfusion, independent of the severity of glomerulosclerosis [29]. Malfunctioning atrophic tubules in the areas of interstitial fibrosis can also affect glomerular function. In either case, correlation of RI with renal tissue damage suggests that RI may be a possible indicator of renal tissue injuries in CKD patients.

Of the clinical indices studied, elevated RI, proteinuria, hypertension, and low eGFR were independent risk factors for CKD progression. This was consistent with a 4-year follow-up study by Sugiura and Wada [17]. Poorer prognosis of patients with RI ≥ 0.7 than those with lower RI was also consistent with other studies showing the potential significance of RI as a prognostic indicator [4, 12–17]. In the normal RI group, despite having heavier proteinuria at renal biopsy, patients who underwent steroid therapy showed a similarly high rate of survival as those who did not undergo steroid therapy. It was speculated that steroid administration contributed to the favorable outcomes through, for example, reduction of proteinuria, and prevention of persistent hypoalbuminemia that could cause hypovolemic renal failure in nephrotic patients. Moreover, the patients in the high-normal RI group showed excellent response to steroids; however, steroid therapy did not significantly improve renal outcome in the patients in the high RI group. Lower eGFR and advanced GS in the high RI group can explain the poor response to steroid treatment. Interestingly, the disease severity reflected by GS had a greater impact on renal prognosis than the disease chronicity estimated from TI lesions. Although the decisions on steroid administration must be carefully taken in each case, RI is considered to be useful not only as a prognostic indicator but also as a non-invasive determinant of indication for steroids.

Finally, increased RI may not always be a result of renal dysfunction, and many cardiovascular factors such as vascular compliance, pulsatility [30, 31], heart rate [32], and the administration of ACE-I [33] or ARB [34] can also affect RI. In this study, although the correlations of RI with clinical and histological indices were not changed even when stratified by the use of ACE-I and/or ARB, associations of RI with age and systolic blood pressure were consistent with other studies showing the impact of arteriosclerosis on RI [6, 35]. In fact, it could be difficult to set an absolute normal RI limit because it can vary with patient age and also with many cardiovascular factors. However, in this study, poor survival of the patients with high-normal range RI (0.65–0.7) without steroid therapy indicated risks of increased RI even within normal limits. Evaluations for arteriosclerosis, such as intimal-medial thickness of the carotid artery and pulse wave velocity, would be favorable in CKD patients with high-normal range RI. Further investigations with a longer follow-up period could help provide a more precise evaluation of the roles of RI.

## 5. Conclusions

RI in CKD patients was considered as a marker of renal function, histological damage, and renal prognosis, and a possible determinant of indication for steroids. In addition, CKD patients with high-normal range RI (0.65–0.70) were also at risk for adverse renal prognosis.

## Figures and Tables

**Table 1:** The correlation coefficients of ultrasonographic measurements with the clinical and histological parameters by univariate analysis (*n* = 202).

	Resistive index	Renal length	Cortex area
Age	0.45**	−0.17*	−0.12
Systolic blood pressure	0.33**	−0.02	0.03
Urinary protein level	0.10	0.06	0.10
Serum creatinine level	0.46**	−0.12	−0.12
eGFR	−0.52**	0.23**	0.20**
Glomerulosclerosis score	0.32**	−0.24**	−0.21**
Arteriolosclerosis score	0.36**	−0.15*	−0.06
Tubulointerstitial damage score	0.43**	−0.16*	−0.15*
CKD stage	0.50**	−0.26**	−0.22**

*P < 0.05, **P < 0.01.

**Table 2:** Comparisons of the clinical and histological data of patients with different levels of RI at renal biopsy by multiple univariate analysis.

	Normal RI group	High-normal RI group	High RI group
	RI < 0.65	0.65 ≤ RI < 0.70	RI ≥ 0.70
	(*n* = 120)	(*n* = 43)	(*n* = 39)
Age	42.5 ± 1.4	50.5 ± 2.7*	62.6 ± 2.1^∗∗††^
Systolic blood pressure (mmHg)	121 ± 1	127 ± 3	136 ± 3**
Urinary protein level (g/gCr)	3.5 ± 0.4	3.2 ± 0.5	5.1 ± 1.1
eGFR (mL/min/1.73 m^2^)	73.4 ± 2.3	50.6 ± 4.1**	38.4 ± 4.5^∗∗†^
GS score	1.2 ± 0.1	1.6 ± 0.2	2.3 ± 0.2^∗∗†^
AS score	0.7 ± 0.1	1.0 ± 0.1	1.2 ± 0.1**
TI score	1.2 ± 0.1	1.8 ± 0.1**	2.2 ± 0.1**
Resistive index	0.576 ± 0.004	0.667 ± 0.002**	0.761 ± 0.009^∗∗††^
Renal length (cm)	10.8 ± 0.1	10.7 ± 0.1	10.3 ± 0.1**

*P < 0.05 versus normal RI group, **P < 0.01 versus normal RI group, ^†^ *P* < 0.05 versus high-normal RI group, ^††^ *P* < 0.01 versus high-normal RI group.

**Table 3:** The hazard ratios of possible risk factors for CKD progression as determined by univariate and multivariate analyses using the Cox proportional hazard model (n = 202).

	Univariate analysis			Multivariate analysis		
	Hazard ratio	95% CI	*P* value	Hazard ratio	95% CI	*P* value
Age ≥ 60	4.67	2.03–10.77	<0.001	2.60	1.09–6.22	0.031
Gender: male	1.07	0.49–2.37	0.861			
Systolic BP ≥ 140 mmHg	4.62	2.11–10.14	<0.001			
Urinary protein > 1.5 g/gCr	18.64	2.52–138.00	0.004	7.76	1.00–60.14	0.049
eGFR < 50 mL/min/1.73 m^2^	14.80	4.40–49.74	<0.001	5.12	1.48–17.70	0.010
GS score ≥ 2	31.99	7.53–135.97	<0.001	9.49	2.02–44.50	0.004
AS score ≥ 1	7.42	2.78–19.79	<0.001			
TI score ≥ 2	14.90	4.45–49.83	<0.001	3.20	0.89–11.46	0.074
Resistive index ≥ 0.7	5.83	2.65–12.85	<0.001			
Renal length < 9 cm	2.33	0.31–17.24	0.409	3.94	0.47–32.96	0.207
